# Benefits of testing in both bio-secure and production environments in genomic selection breeding programs for commercial broiler chicken

**DOI:** 10.1186/s12711-018-0430-x

**Published:** 2018-11-03

**Authors:** Thinh T. Chu, Setegn W. Alemu, Elise Norberg, Anders C. Sørensen, John Henshall, Rachel Hawken, Just Jensen

**Affiliations:** 1 Department of Molecular Biology and Genetics, Center for Quantitative Genetics and Genomics, Aarhus University, 8830 Tjele, Denmark; 2 Wageningen University and Research Animal Breeding and Genomics, 6709 PG Wageningen, The Netherlands; 3 Department of Animal and Aquacultural Sciences, Norwegian University of Life Sciences, 1432 Ås, Norway; 4 Cobb-Vantress Inc., Siloam Springs, AR 72761-1030 USA

## Abstract

**Background:**

A breeding program for commercial broiler chicken that is carried out under strict biosecure conditions can show reduced genetic gain due to genotype by environment interactions (G × E) between bio-secure (B) and commercial production (C) environments. Accuracy of phenotype-based best linear unbiased prediction of breeding values of selection candidates using sib-testing in C is low. Genomic prediction based on dense genetic markers may improve accuracy of selection. Stochastic simulation was used to explore the benefits of genomic selection in breeding schemes for broiler chicken that include birds in both B and C for assessment of phenotype.

**Results:**

When genetic correlations ($r_{g}$) between traits measured in B and C were equal to 0.5 and 0.7, breeding schemes with 15, 30 and 45% of birds assessed in C resulted in higher genetic gain for performance in C compared to those without birds in C. The optimal proportion of birds phenotyped in C for genetic gain was 30%. When the proportion of birds in C was optimal and genotyping effort was limited, allocating 30% of the genotyping effort to birds in C was also the optimal genotyping strategy for genetic gain. When $r_{g}$ was equal to 0.9, genetic gain for performance in C was not improved with birds in C compared to schemes without birds in C. Increasing the heritability of traits assessed in C increased genetic gain significantly. Rates of inbreeding decreased when the proportion of birds in C increased because of a lower selection intensity among birds retained in B and a reduction in the probability of co-selecting close relatives.

**Conclusions:**

If G × E interactions ($r_{g}$ of 0.5 and 0.7) are strong, a genomic selection scheme in which 30% of the birds hatched are phenotyped in C has larger genetic gain for performance in C compared to phenotyping all birds in B. Rates of inbreeding decreased as the proportion of birds moved to C increased from 15 to 45%.

## Background

In commercial broiler chicken breeding programs, purebred lines are kept under strict bio-secure environmental conditions (B) to avoid the risk of losing the lines and to prevent spread of diseases [1]. In contrast, birds in a commercial production environment (C) live under less strict hygienic conditions and diseases can reduce performance, death, or dysfunction of birds. For example, diseases caused by pathogenic *mycoplasma* are chronic problems in many commercial poultry flocks [2] but these pathogens are completely eradicated in very big commercial breeding programs such as those of Aviagen and Cobb-Vantress [1]. The differences between environments B and C can affect phenotypic expression of traits and change the genetic ranking of breeding birds such that the best individual based on performance in B might not be the best based on performance in C, i.e. genotype by environment interactions (G × E) are expected in this situation. G × E interactions for B versus C have been reported for a number of traits [3–6]. Kapell et al. [4] reported substantial G × E interactions for bodyweight and foot-pad dermatitis; they found that genetic correlations ($r_{g}$) between traits measured in B and C ranged from 0.46 to 0.69 for body weight and from 0.78 to 0.82 for foot-pad dermatitis. N’Dri et al. [6] found that $r_{g}$ for performance in B versus C ranged from 0.74 to 0.82 for body weight and from 0.84 to 0.93 for meat quality traits. Long et al. [5] and Ye et al. [3] also found significant G × E interactions for body weight, mortality, and other performance traits measured in B and C. Thus, a breeding program that is carried out under disease-free conditions, i.e. B, is expected to show reduced genetic gain due to G × E interactions, since only gains obtained in C have substantial economic value.

To improve the performance of commercial animals in the presence of G × E interactions, the classical method uses sib-testing for phenotypes in both B and C environments and pedigree-based best linear unbiased prediction (BLUP) for prediction of breeding values. This method has been used successfully in pig and cattle breeding programs [7–9]. In these studies, a fixed number of animals in B was assumed, with no limit on the number of animals in C. The result was a higher genetic gain when phenotypes were assessed in both B and C environments. However, when $r_{g}$ was 0.9, the extra genetic gain was small and a large amount of information from C was necessary to increase genetic gains significantly. These studies did not investigate situations in which the number of animals available for phenotyping is limited.

In broiler chicken breeding programs, the number of hens mated to a rooster is limited and facilities only allow for a limited number of offspring per hen to be hatched at the same time. In addition, birds in C cannot be used as selection candidates because they cannot be brought back to a B environment due to bio-security restrictions and are, therefore, used only as sources of information for relatives (sibs) in B. In the classical method of sib testing using pedigree, the accuracy of prediction of breeding values for selection candidate birds in B is low due to lack of information on Mendelian sampling terms. For these situations, genomic prediction based on dense marker genotypes can be an interesting option due to better modeling of relationships between individuals and better prediction of the Mendelian sampling terms [10, 11].

Several studies have shown that accuracy of selection can be improved considerably by genomic selection using high-density markers [10, 12]. Genomic selection applied in a pig breeding scheme that combines information of performance from purebreds and crossbreds can significantly increase genetic gain and reduce rates of inbreeding compared to a scheme that uses performance from purebreds only [13]. Modeling traits that are expressed in purebreds and crossbreds is similar to the G × E modeling of a trait expressed in B and C environments. In another deterministic simulation study, van Grevenhof and van der Werf [14] investigated the impact of the proportion of purebred versus crossbred animals in the reference population, the $r_{g}$ between purebred and crossbred traits, and economic weight on performance of purebreds versus crossbreds on genetic gain. They showed that with $r_{g}$ equal to 0.5 and 0.7, increasing the proportion of crossbreds in the reference population from 0 to 100% increased genetic gain of the breeding program, but with an $r_{g}$ of 0.9, inclusion of crossbred animals in the reference population reduced genetic gain. However, to our knowledge, no studies have explored genomic selection breeding programs using sib testing in B and C for broiler chickens.

The proportion of birds phenotyped in B versus C and the level of G × E interaction are important factors to consider in designing broiler chicken breeding schemes [7–9]. Birds in C provide information on animal performance in C but, given the limited number of birds hatched in a selection round, a high proportion of birds in C would reduce selection intensity among selection candidates that remain in B. Therefore, the key to improve genetic gain is to find the best compromise between selection intensity among selection candidates and phenotypes for the target environment. The level of G × E interaction also affects the optimum design of breeding schemes. The genetic correlation between trait in B versus C represents the magnitude of the G × E interaction, and different heritabilities for traits in B versus C can be also important.

The objective of this stochastic simulation study was to compare genomic selection broiler chicken breeding schemes when all selection candidates are kept in a B environment or a proportion of the birds hatched are phenotyped in a C environment. Three factors were investigated: (1) the proportion of birds in B *versus* C; (2) the genetic correlation between the trait measured in B and C (G × E); and (3) the heritability of the trait assessed in C. In addition, sensitivity analyses were carried out to investigate the effects of genotyping strategy and breeding population structure.

## Methods

### Breeding schemes

The breeding schemes were simulated in three stages: (1) generation of a historical base population; (2) simulation of the previous selection programs based on pedigree BLUP with phenotype testing in B only; and (3) application of genomic selection with birds in C and/or B environments for phenotype testing. The historical population (stage 1) was simulated using QMSim [15], while the second and third stages were simulated using the stochastic simulation program ADAM [16].

The simulated genome consisted of 26 chromosomes, with a length ranging from 5 to 195 cM and a total length of 916 cM, representing the major chromosomes in chicken. In the first historical generation, approximately 1000 markers and 150 quantitative trait loci (QTL) per cM was simulated, and each marker and QTL had two alleles with an equal frequency of 0.5. The population was simulated for 950 historical generations in order to establish mutation-drift equilibrium [12]. Over the 950 generations, the population was gradually expanded in size from 1100 to 2400 animals with equal numbers of individuals from both sexes. The population had random mating with no selection or migration. A recurrent mutation rate of 2 × 10^−5^ was simulated for both markers and QTL. In descendants, markers and QTL were inherited from their parents following standard principles of Mendelian inheritance and allowing for recombination [17], which was sampled from a Poisson distribution with scale parameter $\lambda$ = 1 for a 100 cM region. Positions of the recombinations along each chromosome were drawn from a uniform distribution. From the historical population, a base population was created, in which each individual had 40 k neutral marker loci and 2 k QTL, which were randomly drawn from segregating loci with a minor allele frequency of at least 0.05 and were, therefore, randomly distributed along the genome.

The simulated breeding schemes had overlapping generations and in each generation, selection was applied over several time steps. A time step is a selection round, in which offspring are born and tested for phenotypes, and selection is applied. A generation was equivalent to 6.5 time steps. At each time step, a parental group of 16 roosters and 160 hens were randomly mated to produce 1280 offspring birds with 8 offspring per hen. Parents produced offspring for several consecutive time steps. The sex ratio among the offspring was 1:1. From the common base population, birds were chosen randomly to be parents from time steps 1 to 5. In time steps 6 to 8, the selected birds from time steps 1 to 3 were sufficiently mature to be parents, but the remaining parents were from the base population to make up the parental group of 16 roosters and 160 hens within a time step. From time step 9 onwards, the parents were no longer from the base population, but were selected in previous time steps. Different genetic parameters were used to simulate breeding values and phenotypes for birds in the base population. The breeding schemes were simulated for 40 time steps.

In the first 20 time steps, all 1280 birds hatched in each time step were phenotyped in the B environment only and all birds were selection candidates. Selection during this stage was based on pedigree-based BLUP estimated breeding values (EBV) from records in B only. This stage was to mimic the situation of breeding programs in which broiler chicken are selected for a certain period using records in B and was the same for all simulated breeding schemes. From time step 21 to 40, the 1280 birds hatched in each time step were all genotyped and allocated to either the B or C environment for phenotype testing. The number of birds in B or C depended on the scenario of the breeding schemes. Thus, the birds in C were siblings of birds in B. After genotyping and assessment of the phenotype, single-step GBLUP (ssGBLUP) models [18, 19] were used in each time step to estimate the genomic estimated breeding value (GEBV) for all birds. Instead of GBLUP, ssGBLUP was used to exploit all pedigree and phenotype information of birds from the previous time steps 1 to 20 when genomic information was not available. Based on GEBV rankings, breeding parents were always selected from birds in B; due to bio-security restriction, birds in C were not candidates for selection.

### Trait simulation

In a broiler-breeding program, the overall breeding goal includes a number of traits with different economic weights. However, for simplification, only a growth performance type of trait was considered, which is the primary trait in the breeding goal for all broiler-breeding programs [1]. The simulated trait expressed in B and C environments was similar to growth performance in the two environments, and thus its genetic parameters were simulated based on parameters for growth performance in B and C from studies on broiler chicken [4, 6, 20, 21]. The trait expressed in the B environment was defined as the B trait and the trait expressed in the C environment was defined as the C trait.

Theoretically, G × E interactions can result in different ranking of breeding values of birds in B and C, different heritabilities, and genetic variances in each environment [22]. In this simulation, only the first two effects were accounted for because G × E interaction was modelled through $r_{g}$ and the heritability of the C trait. The mean (= 0) and genetic variance (= 1) were assumed to be identical in B and C because heterogeneity of genetic variance does not change rankings between selection candidates when the candidates are located in a single environment, their sibs are in another environment, and performances in the two environments are treated as correlated traits [23]. Non-additive genetic effects were not included in the simulation.

The phenotype of the trait expressed in B or C for the $i$th bird in the base population, $y_{i}$ was calculated as $y_{i} = g_{i} + e_{i}$, where $g_{i}$ is the true breeding value (TBV) of the $i$th bird in the base population for a phenotypic record in B or C, and $e_{i}$ is the environmental term for a phenotypic record in B or C. Each animal had TBV for both B and C traits, which were calculated based on genotype at 2000 QTL. The effects of QTL $$\left[ {\begin{array}{*{20}c} {\alpha_{B} } \\ {\alpha_{C} } \\ \end{array} } \right]$$ were randomly sampled and then scaled to achieve an initial genetic covariance matrix of $$\left[ {\begin{array}{*{20}c} 1 & {r_{g} } \\ {r_{g} } & 1 \\ \end{array} } \right]$$ in the base population. All genetic variance and covariance was explained by additive QTL variance and covariance. During the simulation of breeding scenarios, the effect of a QTL was kept constant, but the allele frequency at each QTL could change due to selection and drift.

The environmental terms for the B and C traits were drawn from a random normal distribution $N\left[ {0, \left( {1 - h^{2} } \right)/h^{2} } \right]$, where $h^{2}$ is the heritability of the B and C traits, respectively. Environmental variance was kept constant through the simulations. Environmental covariance between B and C traits was 0 since each bird has a phenotypic record in only one environment.

### Selection criteria

For selection in time steps 21 to 40, the breeding goal had an economic value of 1 for performance of birds in C and an economic value of 0 for performance of birds in B. During time steps 1 to 20, to emulate the previous breeding program, the selection index had an economic value of 1 for performance of birds in B and 0 for performance of birds in C.

Estimated breeding values were based on the following bivariate mixed linear model:

1 $$\left[ {\begin{array}{*{20}c} {{\mathbf{y}}_{{\mathbf{B}}} } \\ {{\mathbf{y}}_{{\mathbf{C}}} } \\ \end{array} } \right] = \left[ {\begin{array}{*{20}c} {{\mathbf{X}}_{{\mathbf{B}}} } & 0 \\ 0 & {{\mathbf{X}}_{{\mathbf{C}}} } \\ \end{array} } \right]\left[ {\begin{array}{*{20}c} {{\mathbf{b}}_{{\mathbf{B}}} } \\ {{\mathbf{b}}_{{\mathbf{C}}} } \\ \end{array} } \right] + \left[ {\begin{array}{*{20}c} {{\mathbf{Z}}_{{\mathbf{B}}} } & 0 \\ 0 & {{\mathbf{Z}}_{{\mathbf{C}}} } \\ \end{array} } \right]\left[ {\begin{array}{*{20}c} {{\mathbf{g}}_{{\mathbf{B}}} } \\ {{\mathbf{g}}_{{\mathbf{C}}} } \\ \end{array} } \right] + \left[ {\begin{array}{*{20}c} {{\mathbf{e}}_{{\mathbf{B}}} } \\ {{\mathbf{e}}_{{\mathbf{C}}} } \\ \end{array} } \right],$$

where ${\mathbf{y}}_{{\mathbf{B}}}$ and ${\mathbf{y}}_{{\mathbf{C}}}$ are vectors of phenotypic records of birds in B and C; ${\mathbf{b}}_{{\mathbf{B}}}$ and ${\mathbf{b}}_{{\mathbf{C}}}$ are vectors of the fixed effect of time step for records in B and C; ${\mathbf{g}}_{{\mathbf{B}}}$ and ${\mathbf{g}}_{{\mathbf{C}}}$ are vectors of breeding values of the B and C traits; ${\mathbf{X}}_{{\mathbf{B}}}$ and ${\mathbf{Z}}_{{\mathbf{B}}}$ and ${\mathbf{X}}_{{\mathbf{C}}}$ and ${\mathbf{Z}}_{{\mathbf{C}}}$ are incidence matrices associating fixed effects and breeding values to the phenotypic records in B and C; ${\mathbf{e}}_{{\mathbf{B}}}$ and ${\mathbf{e}}_{{\mathbf{C}}}$ are vectors of random residuals in B and in C, respectively. Model (1) assumed $$\left[ {\begin{array}{*{20}c} {{\mathbf{e}}_{{\mathbf{B}}} } \\ {{\mathbf{e}}_{{\mathbf{C}}} } \\ \end{array} } \right]$$ ~ *MVN* $$\left[ {\begin{array}{*{20}c} 0 \\ 0 \\ \end{array} \varvec{ }, \left( {\begin{array}{*{20}c} {{\mathbf{I}}_{{\mathbf{B}}} \sigma_{eB}^{2} } & 0 \\ 0 & {{\mathbf{I}}_{{\mathbf{C}}} \sigma_{eC}^{2} } \\ \end{array} } \right)} \right]$$, where ${\mathbf{I}}_{{\mathbf{B}}}$ and ${\mathbf{I}}_{{\mathbf{C}}}$ are identity matrices corresponding to birds in B and C environments; $\sigma_{eB}^{2}$ and $\sigma_{eC}^{2}$ are environmental variances of B and C traits, respectively.

For time steps 1 to 20, breeding values were estimated using bivariate model (1) with the pedigree-based BLUP approach [24], although there were no phenotypic records for the C trait. In the BLUP model, breeding values were assumed to follow a multivariate normal distribution $MVN\left[ {0,\varvec{ }{\mathbf{A}} \otimes {\mathbf{V}}_{{\mathbf{g}}} } \right]$, where ${\mathbf{A}}$ is the matrix of additive genetic relationships based on pedigree; ${\mathbf{V}}_{{\mathbf{g}}}$ is the 2 × 2 genetic covariance matrix of the B and C traits; $\otimes$ is the Kronecker product. The pedigree relationship matrix was constructed from pedigree traced back to the base population.

For time steps 21 to 40, breeding values were estimated from the bivariate model (1) using the ssGBLUP approach [18, 19], which assumes that $$\left[ {\begin{array}{*{20}c} {{\mathbf{g}}_{{\mathbf{B}}} } \\ {{\mathbf{g}}_{{\mathbf{C}}} } \\ \end{array} } \right]\,\sim\,MVN\left[ {0, {\mathbf{H}} \otimes {\mathbf{V}}_{{\mathbf{g}}} } \right]$$, where ${\mathbf{H}}$ is a combined matrix of the pedigree relationship matrix ${\mathbf{A}}$ and a genomic relationship matrix, with a weight of 0.25 [18, 19] on pedigree relationships. The genomic relationship matrix was constructed based on marker data [25].

In each time step, EBV or GEBV were predicted for all individuals after all records in that time step were obtained. Both BLUP and ssGBLUP models used the true genetic variance components for prediction of breeding values. Computations were carried out using the DMU5 module of DMU package [26]. The prediction in each time step used all information (phenotypes, genomic data and pedigree) of all individuals since time step 1. Thus, although all birds were genotyped in time steps 21 to 40, ssGBLUP was used in order to use the phenotypic records from time steps 1 to 20. Selection of birds for use as parents was carried out right after genetic evaluation, although they were not yet sexually mature.

### Factors investigated

The factors investigated in this study were the genetic correlation ($r_{g}$) between trait records obtained in B and C, heritability of the trait in C and the proportions of birds that were kept in B or transferred to C (Table 1). Parameters $r_{g}$ and heritability were used for trait simulation of birds in the base population. On average, selection intensity for breeding schemes with 0, 15, 30 and 45% birds transferred to C was 2.82, 2.77, 2.70 and 2.62, respectively, for males, and 1.97, 1.90, 1.81 and 1.69, respectively, for females. Combining these three factors yielded 36 simulated scenarios. Schemes without birds phenotyped in C included nine scenarios; the remaining 27 scenarios had a proportion of the birds transferred to the C environment.

**Table 1:** Levels of investigated factors in the simulated breeding programs

Investigated factors	Levels
Proportion of birds transferred to C (%)	0; 15; 30; 45
Heritability in B environment	0.28
Heritability in C environment	0.15; 0.25; 0.35
Genetic correlation ($r_{g}$) between traits measured in B and C	0.5; 0.7; 0.9

### Sensitivity analysis

In the main simulation study, we assumed that all birds were genotyped when genomic selection was introduced in the breeding program. To ensure the general validity of our results, extra simulations were carried out to investigate sensitivity when not all birds were genotyped and/or the number of offspring per hen increased. The breeding programs used in the sensitivity analysis were similar to those described previously except for a few modifications, as specified in Table 2. For the sensitivity analysis, in all cases, only one level of G × E interaction was analyzed, i.e. $r_{g}$ equal to 0.7 and heritability of the C trait equal to 0.15. In each time step, a random 50% of the total number of birds that hatched in each time step were genotyped. In sensitivity analysis simulation 1 (SS1), only 50% of the birds in B and C were genotyped and the number of offspring per hen in each time step was equal to 8 or 10. Thus, SS1 included eight scenarios that had 1200 or 1600 birds with 0, 15, 30 and 45% birds in C. In sensitivity analysis simulation 2 (SS2), breeding schemes with 15 and 30% of the birds in C differed in the proportion of genotypes allocated to birds in B versus C. The number of offspring per hen was 8 in each time step.

**Table 2:** Sensitivity analysis 1 and 2 simulating breeding schemes with 0, 15, 30 and 45% of birds in C (P0, P15, P30 and P45), using 8 (H8) or 10 (H10) offspring per hen hatched for phenotype testing, and allocating 15, 30, 45 and 60% of total genotyping to birds in C (GC15, GC30, GC45 and GC60)

Scenario	Total number of birds hatched for phenotyping	Total number of genotyped birds	Number of birds moved to C (% of birds hatched)	Number of birds in C genotyped (% of total genotyping)
Sensitivity analysis 1				
H8-P0	1280	640	0 (0%)	0 (0%)
H8-P15	1280	640	192 (15%)	96 (15%)
H8-P30	1280	640	384 (30%)	192 (30%)
H8-P45	1280	640	576 (45%)	288 (45%)
H10-P0	1600	800	0 (0%)	0 (0%)
H10-P15	1600	800	240 (15%)	120 (15%)
H10-P30	1600	800	480 (30%)	240 (30%)
H10-P45	1600	800	720 (45%)	360 (45%)
Sensitivity analysis 2				
P15-GC15	1280	640	192 (15%)	96 (15%)
P15-GC30	1280	640	192 (15%)	192 (30%)
P30-GC15	1280	640	384 (30%)	96 (15%)
P30-GC30	1280	640	384 (30%)	192 (30%)
P30-GC45	1280	640	384 (30%)	288 (45%)
P30-GC60	1280	640	384 (30%)	384 (60%)

### Simulation outputs

For each scenario, 50 replicates were simulated. For each replicate, genetic merit ($G_{t}$) at time step $t$ was the average TBV of all birds hatched in time step $t$ for the C trait. The difference between genetic merits at time steps 31 ($G_{31}$) and 40 ($G_{40}$) was used to compute the rate of genetic gain per time step $\left( {\Delta G} \right)$: $\left( {\Delta G} \right) = \left( {G_{40} - G_{31} } \right)/\left( {40 - 31} \right)$.

The inbreeding coefficient of each individual was the proportion of homozygous identical-by-descent markers for the individual [27]. The average inbreeding coefficient $F_{t}$ at time step $t$ was equal to the average of the inbreeding coefficients of the 1280 individual birds hatched at time step $t$. For comparison of our findings to those of other studies, rate of inbreeding per generation was used instead of rate of inbreeding per time step. Therefore, in the calculation of the inbreeding coefficient, time step $t$ was translated to its corresponding generation. The rate of inbreeding per generation $\left( {{{\Delta }}F} \right)$ [28] for a replicate was computed as ${{\Delta }}F \left( \% \right) = \left( {1 - e^{\beta } } \right)*100$, where $\beta$ is the slope of the linear regression of ${ \ln }(1 - F_{t)}$ on generation corresponding to time steps 31 to 40.

The accuracy of GEBV was computed as the correlation between GEBV and TBV of the C trait for all B birds hatched at time step 36, using the GEBV obtained during that time step. Birds selected at time step 36 were the last selected parents that produced offspring at time step 40.

### Data analyses

${{\Delta }}G$ and ${{\Delta }}F$ for each replicate were used for comparison of scenarios in the main simulation study, whereas only ${{\Delta }}G$ was used to assess differences between scenarios in the sensitivity analysis. Descriptive statistics and standard ANOVA were used. Comparison tests for significance using Tukey’s HSD (honest significant difference, P < 0.05) were used. For accuracy of ssGBLUP prediction, only the means are reported.

In the main study, three factors were included in the ANOVA model: the proportion of birds in C, $r_{g}$, and heritability of the C trait. Their main effects and all two- and three-factor interactions were assessed. In the sensitivity analysis simulations, SS1 had eight scenarios, while SS2 had six scenarios. Rates of genetic gain for the eight SS1 scenarios and the four corresponding scenarios of the main study that had the same $r_{g}$ and heritability were combined for analysis using a two-way ANOVA model, which included the number of birds genotyped, the number of offspring per hen (three levels), and the proportion of birds in C (four levels). For SS2, a one-way ANOVA model was applied to compare six scenarios.

## Results

### Rate of genetic gain

**Fig. 1:**
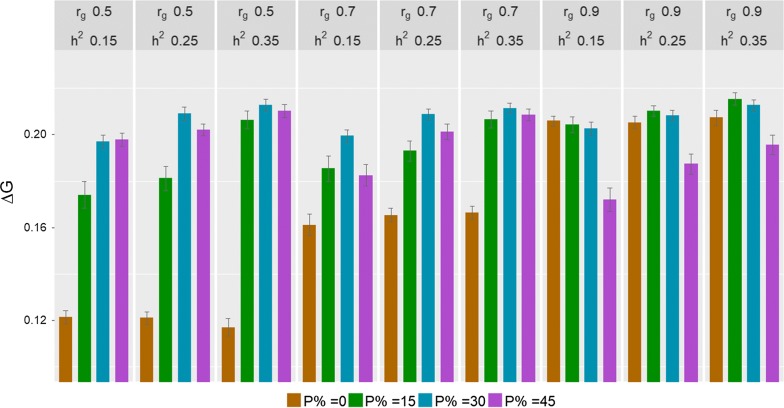
Genetic gain per time step ($\Delta G$) (mean over 50 replicates ± standard error) of scenarios with different proportions of birds in C (P %) for different levels of the genetic correlation ($r_{g}$) between the B and C traits and of heritability ($h^{2}$) of the C trait

The three-factor interaction between the proportion of birds in C, $r_{g}$, and the heritability of the C trait on ${{\Delta }}G$ was not significant (P = 0.099). Significant two-factor interaction effects on ${{\Delta }}G$ were found between the proportion of birds in C and $r_{g}$ (P < 0.001) and between the proportion of birds in C and heritability (P < 0.001). The interaction between $r_{g}$ and heritability did not have a significant effect on ${{\Delta }}G$ (P = 0.562). Figure 1 shows the genetic gain of breeding schemes for different $r_{g}$ and heritability of the trait recorded in C.

The effect of the two-way interaction between the proportion of birds in C and $r_{g}$ on $\Delta G$ was significant. With an $r_{g}$ of 0.5 and 0.7, ${{\Delta }}G$ of breeding schemes without birds in C was significantly lower than that of schemes with birds in C. On average, schemes without birds in C had a ${{\Delta }}G$ of 0.116 and 0.164 per time step with $r_{g}$ equal to 0.5 and 0.7, respectively, while schemes with birds in C had ${{\Delta }}G$ of 0.199 and 0.200 with $r_{g}$ equal to 0.5 and 0.7, respectively. Among the schemes with birds in C, with $r_{g}$ of 0.5 and 0.7, ${{\Delta }}G$ of the schemes with 30 and 45% birds in C was significantly higher than ${{\Delta }}G$ of the scheme with 15% birds in C (P < 0.05). With an $r_{g}$ of 0.9, ${{\Delta }}G$ of the schemes with 0, 15 and 30% birds in C were not significantly different from each other (P > 0.05), but they were significantly higher than ${{\Delta }}G$ of the scheme with 45% of birds in C (P < 0.05).

Changes in ${{\Delta }}G$ with increasing $r_{g}$ varied for different proportions of birds in C. Increasing $r_{g}$ increased ${{\Delta }}G$ of schemes without birds in C significantly, and an increase in ${{\Delta }}G$ with increasing $r_{g}$ was also observed for the scheme with 15% birds in C. However, ${{\Delta }}G$ of the scheme with 30% birds in C did not change significantly as $r_{g}$ increased. For the scheme with 45% birds in C, ${{\Delta }}G$ tended to decrease when $r_{g}$ increased. Thus, ${{\Delta }}G$ of schemes with 30% birds in C were similar for different $r_{g}$.

The effect of the interaction between the proportion of birds in C and heritability on ${{\Delta }}G$ was significant. With heritabilities of 0.15 and 0.25, a change in the proportion of birds in C led to significant differences in ${{\Delta }}G$ between schemes with birds in C. With a heritability of 0.35, changes in ${{\Delta }}G$ due to the proportion of birds in C were not significant between schemes with birds in C. More importantly, ${{\Delta }}G$ increased as heritability increased in schemes with birds in C. However, ${{\Delta }}G$ in the scheme without birds in C, as expected, was not affected by heritability of the C trait. On average, ${{\Delta }}G$ of scenarios with heritabilities of 0.15, 0.25 and 0.35 were 0.161, 0.163 and 0.161, respectively, for schemes without birds in C, while ${{\Delta }}G$ was 0.191, 0.200 and 0.210, respectively, for schemes with birds in C.

In addition, the effect of the two-factor interaction between the proportion of birds in C and heritability on ${{\Delta }}G$ suggested that schemes with birds in C had significantly higher ${{\Delta }}G$ than schemes without birds in C regardless of heritability (0.15, 0.25 and 0.35). However, if $r_{g}$ had been not accounted for, these results could be misinterpreted. For example with an $r_{g}$ of 0.9, a change in heritability did not cause differences in ${{\Delta }}G$ among breeding schemes with 0, 15 and 30% birds in C but with heritabilities of 0.25 and 0.35, schemes with 15 and 30% birds in C tended to have higher ${{\Delta }}G$ than schemes without birds in C (P > 0.05). Differences in ${{\Delta }}G$ between breeding schemes with different proportions of birds in C depended on both $r_{g}$ and heritability.

### Rate of inbreeding

The effect of the three-factor interaction between the proportion of birds in C, $r_{g}$, and heritability of the C trait on ${{\Delta }}F$ was not significant (P = 0.445). Significant interactions on ${{\Delta }}F$ were found between the proportion of birds in C and $r_{g}$ (P = 0.005) and between $r_{g}$ and heritability (P = 0.043). The effect of the interaction between the proportion of birds in C and heritability on ${{\Delta }}F$ was not significant (P = 0.085).

**Fig. 2:**
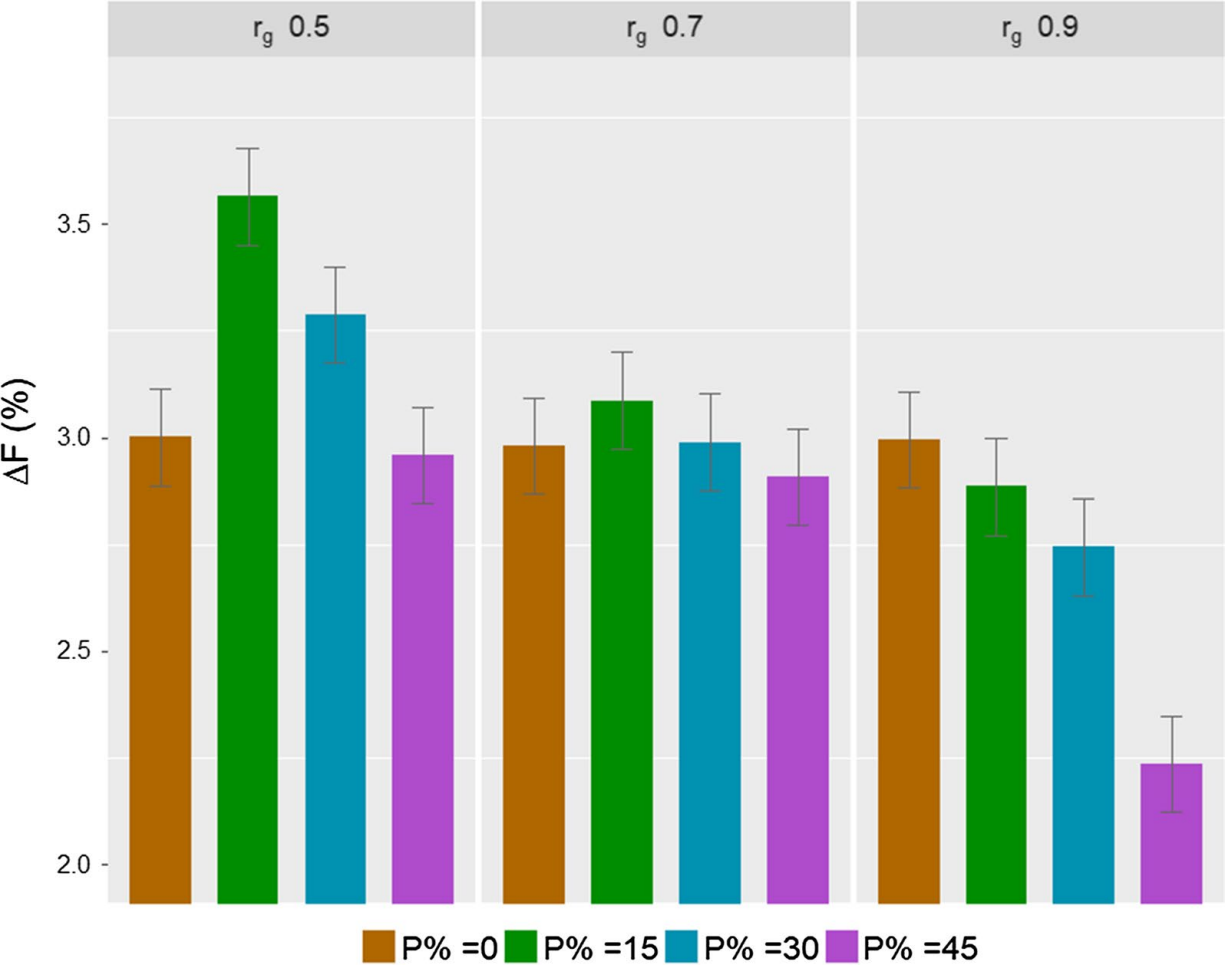
Rate of inbreeding per generation (${{\Delta }}F$%) (mean over 150 replicates ± standard error) of breeding schemes with different proportions of birds in C for different levels of the genetic correlation ($r_{g}$) between the B and C traits

The proportion of birds in C affected ${{\Delta }}F$ differently as $r_{g}$ changed (Fig. 2). On the one hand, with increasing $r_{g}$, ${{\Delta }}F$ of schemes without birds in C did not change. On the other hand, ${{\Delta }}F$ of schemes with birds in C decreased with increasing $r_{g}$. On average, ${{\Delta }}F$ of schemes with $r_{g}$ of 0.5, 0.7 and 0.9 were 3.27, 2.99 and 2.62% per generation, respectively. As the proportion of birds in C increased, ${{\Delta }}F$ decreased. With an $r_{g}$ of 0.5, ${{\Delta }}F$ was lowest for schemes with 0 and 45% birds in C and highest for the scheme with 15% birds in C. With an $r_{g}$ of 0.7, ${{\Delta }}F$ was not significantly different between schemes. With an $r_{g}$ of 0.9, schemes without birds in C had the highest ${{\Delta }}F$, followed by schemes with 15, 30 and 45% birds in C.

**Fig. 3:**
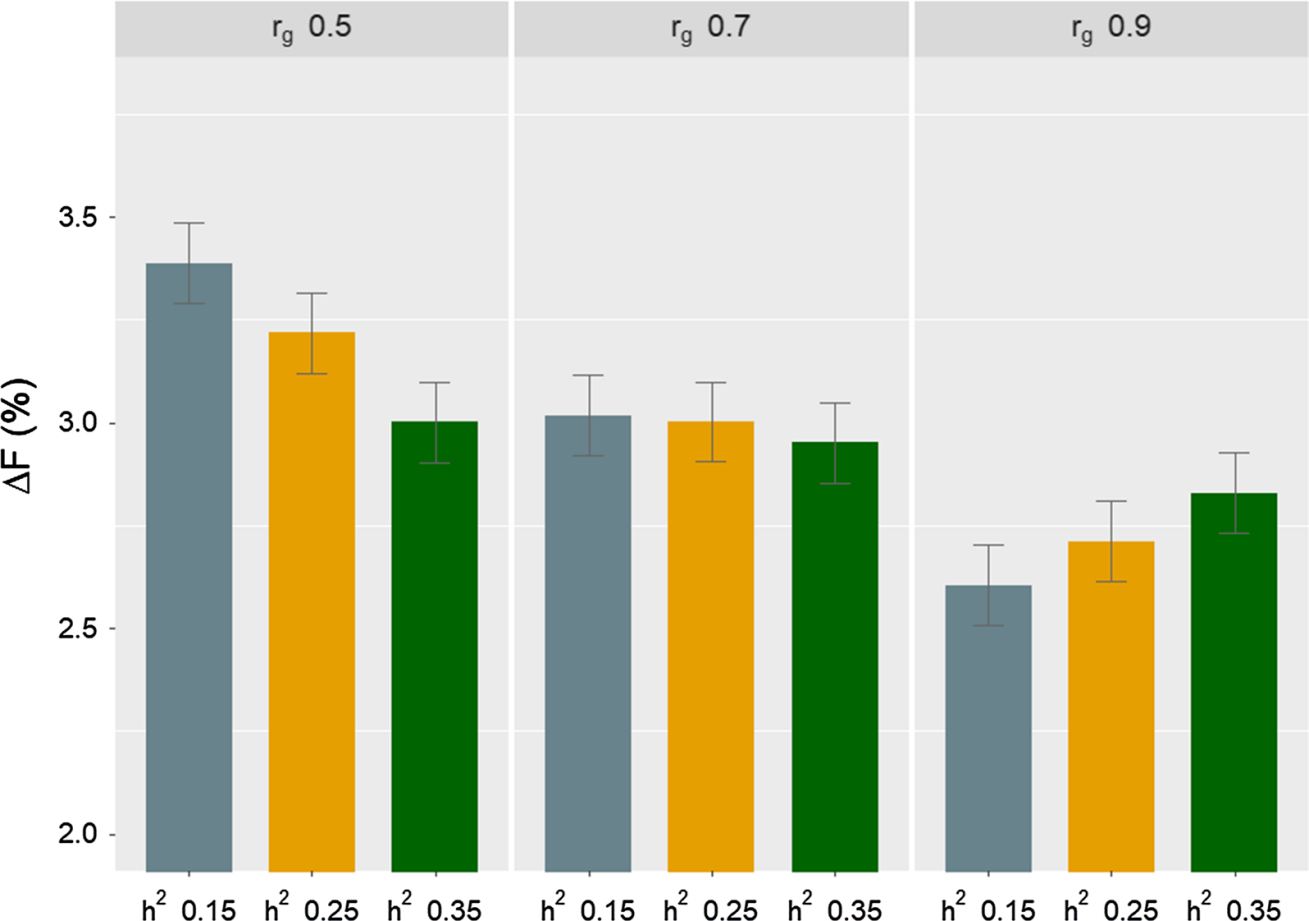
Rate of inbreeding per generation (${{\Delta }}F$%) (mean over 200 replicates ± standard error) for different levels of the genetic correlation ($r_{g}$) between the B and C traits and of the heritability ($h^{2}$) of the C trait

The effect of the interaction between the effects of $r_{g}$ and heritability of C trait on ${{\Delta }}F$ was found to be significant. With an $r_{g}$ of 0.7, there were no significant differences in ${{\Delta }}F$ between all levels of heritability (Fig. 3). With an $r_{g}$ of 0.5, ${{\Delta }}F$ tended to decrease with increasing heritability. With an $r_{g}$ of 0.9, ${{\Delta }}F$ increased as heritability increased.

### Sensitivity analyses

**Fig. 4:**
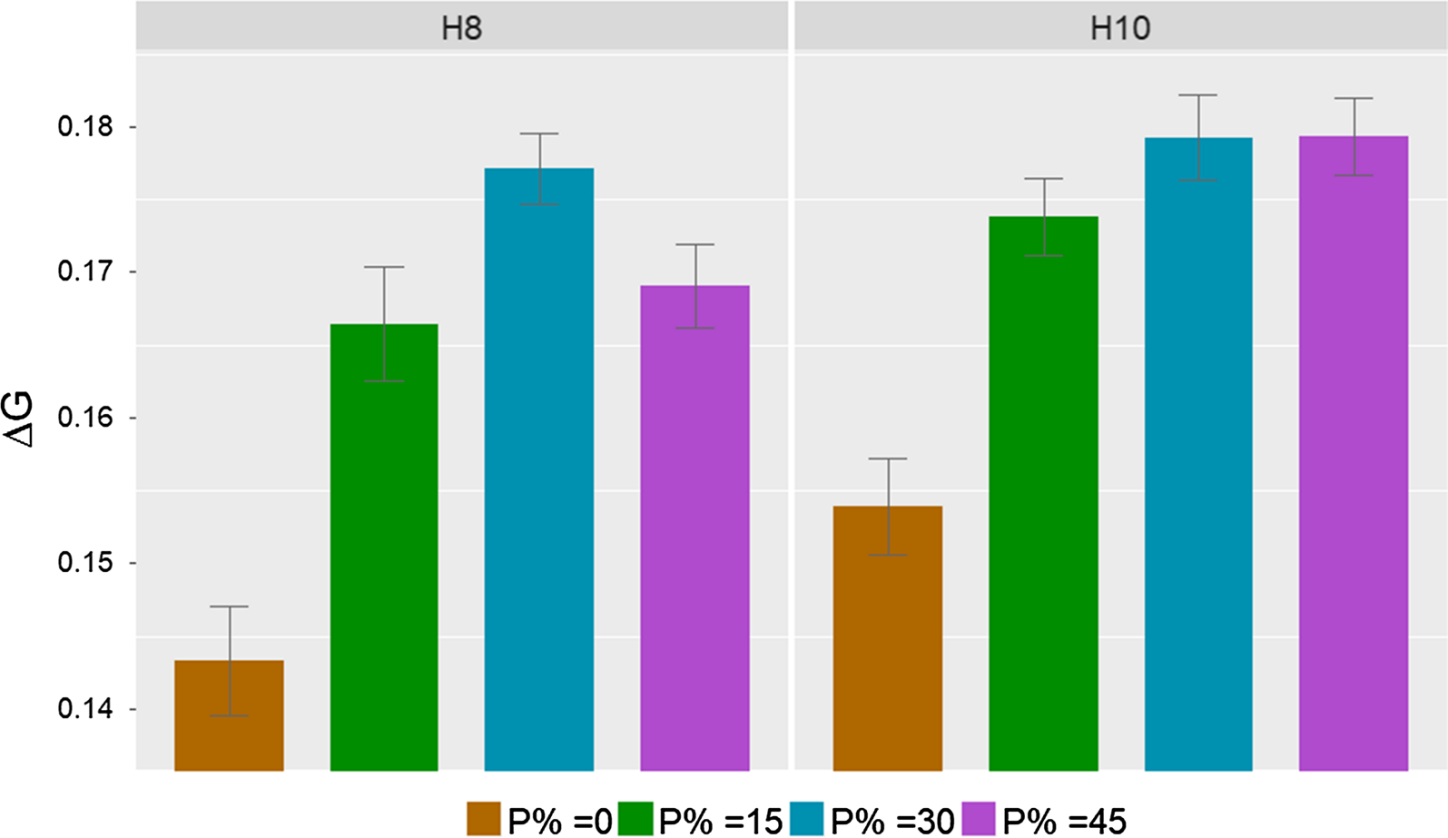
Genetic gain per time step ($\Delta G$) (mean over 50 replicates ± standard error) of sensitivity simulation 1 for breeding schemes with 8 (H8) or 10 (H10) offspring per hen per time step and different proportions of birds in C (P %)

In SS1, breeding schemes with 8 and 10 offspring per hen in each time step had 0, 15, 30 and 45% birds in C when only 50% of birds in B and C were genotyped. We found that the schemes of SS1 had lower ${{\Delta }}G$ than corresponding schemes in the base situation. In SS1, schemes with 8 offspring per hen per time step had lower ${{\Delta }}G$ than schemes with 10 offspring per hen. However, similar to the base situation, schemes without birds in C had the lowest ${{\Delta }}G$ among all breeding schemes investigated in SS1 (Fig. 4). In addition, the scheme with 30% birds in C had the highest ${{\Delta }}G$, followed by schemes with 15 and 45% birds in C when the number of offspring per hen was 8. ${{\Delta }}G$ tended to increase as the proportion of birds in C increased from 0 to 45% when the number of offspring per hen was 10 but the rate of increase in ${{\Delta }}G$ decreased as the proportion of C birds increased. The scheme with 45% of birds in C had the highest ${{\Delta }}G$ when the number of offspring per hen was 10. However, the difference in ${{\Delta }}G$ between schemes with 30 and 45% birds in C was small.

**Fig. 5:**
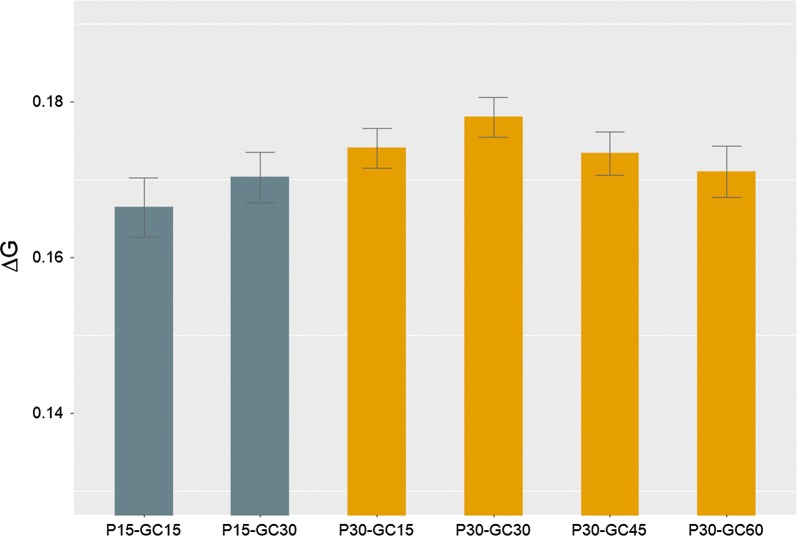
Genetic gain per time step ($\Delta G$) (mean over 50 replicates ± standard error) of sensitivity simulation 3 for breeding schemes with 15 and 30% (P15 and P30) of birds in C and allocating 15, 30, 45 and 60% of genotyping to birds in C (GC15, GC30, GC45 and GC60)

In SS2, genetic gain of breeding schemes with 15 and 30% birds in C that used different genotyping strategies was examined when the number of genotyped birds was kept constant (Fig. 5). For breeding schemes with 15% birds in C, the scheme with 30% of genotyping allocated to birds in C yielded a higher ${{\Delta }}G$ than the scheme with 15% of genotyping allocated to birds in C. For the breeding scheme with 30% birds in C, the strategy with 30% of genotyping allocated to birds in C resulted in the highest ${{\Delta }}G$. For a constant number of genotyped birds, ${{\Delta }}G$ tended to decrease as the proportion of genotyping allocated to birds in C increased from 30 to 60%. Among the six schemes of SS2, ${{\Delta }}G$ was highest in the scheme with 30% birds in C and 30% of genotyping allocated to birds in C.

## Discussion

In this study, we investigated genetic gain (${{\Delta }}G$) and rate of inbreeding (${{\Delta }}F$) in different genomic breeding schemes for broiler chickens with varying levels of G $\times$ E interactions between the bio-secure breeding environment (B) and the commercial environment (C). We investigated schemes with 0, 15, 30 and 45% birds in C. The effects of G $\times$ E interaction were modelled by varying the genetic correlation ($r_{g}$) between the B and C traits (0.5, 0.7 and 0.9) and the heritability of the C trait (0.15, 0.25 and 0.35). Sensitivity analyses were also carried out to further investigate the effects of genotyping strategy and of the number of offspring per hen.

### Rate of genetic gain

Genetic gain in the main study was influenced by the proportion of birds in B versus C, $r_{g}$ between B and C traits, and heritability of the C trait. The proportion of birds in B versus C has an effect on the accuracy of selection and on selection intensity. Since only the performance in C has an economic value, a higher proportion of birds in C resulted in a higher accuracy of GEBV. For example, when $r_{g}$ was 0.5 and heritability was 0.25, accuracies of GEBV were 0.369, 0.718, 0.777 and 0.804 for 0, 15, 30 and 45% birds in C, respectively. When $r_{g}$ was 0.9 and heritability was 0.25, accuracies of GEBV were 0.745, 0.809, 0.819 and 0.837 for 0, 15, 30 and 45% birds in C, respectively. Even with an $r_{g}$ of 0.9, increasing the proportion of birds in C resulted in increases in the accuracy of EBV. Therefore, genetic gain of a breeding program can be improved by producing records in the C environment if there is a significant level of G $\times$ E interaction in the population. This was illustrated also by Bijma and Arendonk [7] and Mulder and Bijma [9], who found that genetic gain was improved with extra information from C when $r_{g}$ was less than 1. However, given limited hatching and reproductive capacities, increasing the number of birds in C reduces selection intensity in B due to a reduction in the number of selection candidates. In other words, there is a trade-off between additional accuracy of selection from records in C and a reduction in selection intensity. In our main study, 30% of the birds in C resulted in the optimal balance between accuracy and selection intensity.

The optimal breeding scheme in terms of the proportion of birds in B versus C depends on the extent of G $\times$ E interaction. Transferring birds from B to C did not improve ${{\Delta }}G$ in all situations. The level of $r_{g}$ changed the accuracy of GEBV in the scenarios investigated. For example, when the proportion of birds in C was 0 and heritability was 0.25, the accuracy of GEBV was equal to 0.369, 0.534 and 0.745 for $r_{g}$ of 0.5, 0.7 and 0.9, respectively. The contribution of records on birds in B to the accuracy of GEBV increased as $r_{g}$ between the two environments increased. In other words, the contribution of records on birds in C relative to the contribution of records on birds in B to the accuracy of GEBV decreased as $r_{g}$ increased, which explains the increase in ${{\Delta }}G$ with increasing $r_{g}$ for schemes without birds in C. The difference in ${{\Delta }}G$ between schemes with and without C birds was also smaller when $r_{g}$ increased or when there was less G $\times$ E interaction. Dekkers [13] concluded that with an $r_{g}$ of 0.7, genomic selection could improve genetic gain if information from records on birds in B and C was combined. However, van Grevenhof and van der Werf [14] implied that with an $r_{g}$ of 0.9, transferring animals from the B to the C environment did not increase genetic gain. Therefore, when the level of G $\times$ E interaction is low ($r_{g}$ = 0.9), transferring birds from B to C is not necessary. Nonetheless, with a low level of G $\times$ E interaction, one possible benefit of including records on birds tested in C is to enable selection for disease resistance, especially diseases that exist in the C environment but not in B [29].

Apart from $r_{g}$, heritability of the C trait had a significant effect on ${{\Delta }}G$ for breeding schemes with birds in C. The level of $r_{g}$ relates to the contribution of records in B to the accuracy of GEBV prediction while the level of the heritability of C trait relates to the contribution of records on birds in C to the accuracy of GEBV. As the heritability of the C trait increases, the contribution of records to the accuracy of GEBV increases. For example, with an $r_{g}$ of 0.7 and 30% of birds in C, accuracies of GEBV were equal to 0.756, 0.793 and 0.827 for heritabilities of 0.15, 0.25 and 0.35, respectively. However, in schemes without birds in C, the heritability of the C trait was not important. Genetic gain in the C trait in schemes without birds in C depends on the amount of genetic variation, selection intensity and $r_{g}$. In our simulations, ${{\Delta }}G$ is in genetic standard deviations and the genetic standard deviation was kept constant as the heritability of the C trait changed. Therefore, no change in ${{\Delta }}G$ was observed in schemes without birds in C as heritability of the C trait increased.

When designing breeding programs, effects of G × E interaction are often modelled through $r_{g}$, while heterogeneous heritability of traits across environments is often not taken into account [7, 9, 13, 14]. The value of $r_{g}$ expresses the magnitude of the G × E interaction but G × E interaction can also result in heritabilities of the B and C traits being different. Heritability of the C trait can be lower or higher than heritability of the B trait. Kapell et al. [4] reported that for three of the four studied pure broiler lines, the C trait had a lower heritability than the B trait for body weight at 5 weeks of age. Both higher and lower heritability for the C trait than for the B trait were also found by N’Dri et al. [6]. Heritability of the B trait can also have a significant effect on the design of a breeding program that takes G × E interactions into account but this was not investigated in our study. The contribution of records on birds in B would increase with increasing heritability of the B trait.

In addition to the proportion of birds in C, $r_{g}$, and heritability of the C trait, sensitivity analyses showed that the amount of genotyping, the number of offspring per hen and genotyping strategies can influence genetic gain of a genomic selection program for broilers. In the sensitivity analysis, only 50% of all birds that hatched were genotyped, $r_{g}$ was 0.7, and heritability of the C trait was 0.15. Genetic gain of the corresponding scenarios in the main study were higher than in the SS1 scenarios, even for SS1 scenarios that had 10 offspring per hen, compared to 8 for the scenarios in the main study, primarily because of the smaller number of birds genotyped in the SS1 scenarios: 640 and 800 for the schemes with 8 and 10 offspring per hen, respectively, compared 1280 birds genotyped in each time step in the main study.

When the number of offspring per hen was 8, relative differences in ${{\Delta }}G$ between the SS1 schemes with 0, 15, 30 and 45% birds in C were similar to those of the main study, and schemes with 30% birds in C had the highest ${{\Delta }}G$. In the latter case, selection intensity was not changed when comparing the SS1 schemes with 8 offspring per hen to the corresponding schemes of the main study, and proportions of birds in B versus C among the schemes were the same as the proportions of birds in B versus C genotyped. When the number of offspring per hen increased from 8 to 10 per time step, schemes with 45% of birds in C had the highest ${{\Delta }}G$. However, the difference between schemes with 30 and 45% of birds in C was very small. This implies that 30% birds in C is close to optimal when the number of offspring per hen is 8 and 10 per time step.

In SS2, ${{\Delta }}G$ of the scheme with 15% of birds in C tended to increase as the number of genotyped birds in C increased, which suggests that a higher proportion of birds in C should be genotyped when the proportion of birds in C is less than optimal. Therefore, the scheme with 15% of birds in C for phenotyping and with all these birds genotyped was expected to yield a higher ${{\Delta }}G$ than the scheme with 30% of birds tested in C and 50% of these birds genotyped. However, the decrease in ${{\Delta }}G$ of the scheme with 15% compared to 30% of birds in C was not compensated by increasing the number of genotyped birds in C and increasing selection intensity.

The scheme with 30% of the birds in C and 30% of genotyping allocated to birds in C has the highest ${{\Delta }}G$ in SS2. Increasing the number of genotyped birds in C increases the amount of information from C. However, when genotyping resources are limited, increasing the number of C birds that are genotyped reduces the number of B birds genotyped. Information “brought” from the C to the B environment is less meaningful as the number of B birds genotyped decreases, which may explain the reduction in ${{\Delta }}G$ as the proportion of genotyping allocated to birds in C increased from 30 to 60%.

A genotyping strategy that was not tested was selective genotyping. Boligon et al. [30] found that a selective genotyping strategy improves the accuracy of GEBV and that animals with the best performance are the most informative. Selective genotyping is possible in broilers because important traits such as body weight and feed efficiency can be measured before sexual maturity. When applying this strategy, it is necessary to consider whether selective genotyping should be applied to birds in B, C, or both. Especially in B, selective genotyping can be advantageous in order to increase genetic gain for a given investment in genotyping.

### Rate of inbreeding

Along with genetic gain, we investigated rates of inbreeding in the schemes of the main study. We found that the proportion of birds in C, $r_{g} ,$ and heritability of the C trait all affected ${{\Delta }}F$. Among the schemes that used records from C, ${{\Delta }}F$ decreased as $r_{g}$ increased or when the proportion of birds in C increased. Heritability of the C trait had different effects on ${{\Delta }}F$ for different levels of $r_{g}$.

Transferring birds from the B to the C environment reduces selection intensity and increases the amount of information obtained from C. Reducing selection intensity reduces ${{\Delta }}F$ because decreasing the number of selection candidates decreases the probability of co-selecting birds from the same family. Increasing the amount of information from C has two opposite consequences on ${{\Delta }}F$. One consequence is an increase in the probability of co-selecting close relatives, which increases ${{\Delta }}F$. For example, a group of closely related selection candidates receives similar information from C and, therefore, the probability of co-selecting close relatives increases. In addition, increasing the proportion of birds in C increases the weight or the contribution of C information to GEBV of birds in B, which increases ${{\Delta }}F$. Another consequence of increasing the amount of information from C is an increase in the accuracy of prediction, especially because genomic information describes relationships between full-sibs better than pedigree information. As the amount of information from C increases, the accuracy of GEBV of birds in B increases and, therefore, the probability of co-selecting close relatives decreases.

An extra simulation was carried out to test the effect on ${{\Delta }}F$ when information from C increases and selection intensity remains constant. Heritability of the C trait was 0.15, and $r_{g}$ was 0.5 or 0.9. The breeding scheme for this simulation was the same as in the main study, except that the number of offspring per hen was varied from 4, to 5, 6, 7 and 8, which is equivalent to 640, 800, 960, 1120 and 1280 birds hatched in each time step. In each time step, 640 birds were in B as selection candidates, and the remaining were transferred to C. With an $r_{g}$ of 0.9, ${{\Delta }}F$ was 2.18, 2.37, 2.49, 2.52 and 2.65 for schemes with 4, 5, 6, 7 and 8 offspring per hen, respectively. With an $r_{g}$ of 0.5, ${{\Delta }}F$ was 2.48, 3.29, 3.15, 2.99 and 2.82 for schemes with 4, 5, 6, 7 and 8 offspring per hen, respectively. These results confirm that increasing information from C has two opposite consequences on ${{\Delta }}F$, as explained above.

When $r_{g}$ is equal to 0.5, the effect of the co-selection on ${{\Delta }}F$ due to using information from C is substantial for schemes with 15 and 30% of birds in C, which leads to higher ${{\Delta }}F$ of these schemes than schemes without birds in C, although the latter, indeed, has the highest selection intensity. As $r_{g}$ increases, information from the B environment has more weight for GEBV prediction, which reduces the probability of co-selection due to using C information. Therefore, changes in $r_{g}$ result in changes in probabilities of co-selection due to using C information. This explains the reduction in ${{\Delta }}F$ of schemes with birds in C as $r_{g}$ increases. Meanwhile, ${{\Delta }}F$ of schemes without birds in C is not affected by a change in $r_{g}$.

As heritability of the C trait increased, the pattern of changes in ${{\Delta }}F$ depended on $r_{g}$ because changing the heritability of the C trait has two opposite consequences on ${{\Delta }}F$. One consequence is that an increase in heritability decreases the weight on information from relatives in BLUP, which reduces the probability of co-selection of relatives [31, 32] and ${{\Delta }}F$. Another consequence of increasing the heritability of the C trait is that it increases the weight of C information, which increases ${{\Delta }}F$. With a low $r_{g}$ of 0.5, these increases in the weight of C information for prediction do not clearly show its effect, but it increases ${{\Delta }}F$ with a high $r_{g}$ of 0.9.

## Conclusions

Genetic gain and rate of inbreeding of genomic breeding schemes for broiler chickens were compared for different degrees of G $\times$ E interaction between breeding (B) and commercial (C) environments. We showed that the proportion of birds in B versus C for a breeding program that maximizes genetic gain depends on the genetic correlation between the trait assessed in B and in C ($r_{g}$), heritability of the trait measured in C, the number of offspring per hen, the amount of genotyping, and the genotyping strategy. With an $r_{g}$ of 0.5 and 0.7, transferring birds from B to the C environment increased genetic gain for the breeding program and 30% of birds assessed in C was optimal. When the proportion of birds in C was optimal (30%) and genotyping efforts were limited, 30% of the genotyping effort allocated to C birds was also the optimal genotyping strategy. When the proportion of birds in C was less than optimal, genotyping more birds in C increased genetic gain. Increasing the proportion of birds in C reduced the rate of inbreeding. The rate of inbreeding of schemes with birds in C increased when $r_{g}$ increased, whereas that of schemes without birds in C did not change. In summary, if G × E interaction is strong ($r_{g}$ equal to 0.5 and 0.7), a genomic selection scheme that evaluates a considerable proportion (30%) of birds in C yields more genetic gain than evaluating all birds in B. In addition, rate of inbreeding decreases as the proportion of birds transferred from B to C increases from 15 to 45%.
